# Activity-based cost analysis of hepatic tumor ablation using CT-guided high-dose rate brachytherapy or CT-guided radiofrequency ablation in hepatocellular carcinoma

**DOI:** 10.1186/s13014-016-0606-x

**Published:** 2016-02-25

**Authors:** D. Schnapauff, F. Collettini, I. Steffen, G. Wieners, B. Hamm, B. Gebauer, M. H. Maurer

**Affiliations:** Department of Radiology, Charité – Universitätsmedizin Berlin, Augustenburger Platz 1, 13353 Berlin, Germany; Department of Radiology, University of Bern, Inselspital, Freiburgstr. 10, 3010 Bern, Switzerland

**Keywords:** CT guidance, High-dose rate brachytherapy, Radiofrequency ablation, Cost analysis, Liver, Minimally invasive treatment

## Abstract

**Purpose:**

To analyse and compare the costs of hepatic tumor ablation with computed tomography (CT)-guided high-dose rate brachytherapy (CT-HDRBT) and CT-guided radiofrequency ablation (CT-RFA) as two alternative minimally invasive treatment options of hepatocellular carcinoma (HCC).

**Materials and methods:**

An activity based process model was created determining working steps and required staff of CT-RFA and CT-HDRBT. Prorated costs of equipment use (purchase, depreciation, and maintenance), costs of staff, and expenditure for disposables were identified in a sample of 20 patients (10 treated by CT-RFA and 10 by CT-HDRBT) and compared. A sensitivity and break even analysis was performed to analyse the dependence of costs on the number of patients treated annually with both methods.

**Results:**

Costs of CT-RFA were nearly stable with mean overall costs of approximately 1909 €, 1847 €, 1816 € and 1801 € per patient when treating 25, 50, 100 or 200 patients annually, as the main factor influencing the costs of this procedure was the single-use RFA probe. Mean costs of CT-HDRBT decreased significantly per patient ablation with a rising number of patients treated annually, with prorated costs of 3442 €, 1962 €, 1222 € and 852 € when treating 25, 50, 100 or 200 patients, due to low costs of single-use disposables compared to high annual fix-costs which proportionally decreased per patient with a higher number of patients treated annually. A break-even between both methods was reached when treating at least 55 patients annually.

**Conclusion:**

Although CT-HDRBT is a more complex procedure with more staff involved, it can be performed at lower costs per patient from the perspective of the medical provider when treating more than 55 patients compared to CT-RFA, mainly due to lower costs for disposables and a decreasing percentage of fixed costs with an increasing number of treatments.

## Key points

- From the perspective of a healthcare provider, CT-guided high-dose rate brachytherapy (CT-HDRBT) of HCC is less expensive to perform than the standard therapy of CT-guided radiofrequency ablation (CT-RFA) when treating only a relatively small number of 55 patients annually
- The cost advantage of CT-HDRBT is mainly based on the much lower cost of disposables compared with the high costs of probes for CT-RFA
- The personnel involvement is higher for CT-HDRBT, but extra staff costs are negligible compared to the costs of disposables for CT-RFA

## Introduction

The use of hepatic tumor ablation has significantly increased during the past two decades, especially for the treatment of hepatocellular carcinoma (HCC) [1, 2]. In the treatment guidelines developed by the Barcelona Liver Clinic Cancer Group (BCLC), tumor ablation is defined as the primary treatment method in patients with very early and early stage HCC (BCLC 0 & A) [3]. As the incidence of HCC is rising and life expectancy in general is increasing, so the amount of patients undergoing tumor ablation will increase further.

Currently radiofrequency ablation (RFA) is the most commonly used ablation method [4]. However, RFA has several limitations, like size limitations, the heat-sink effect when tumor masses are close to cooling vessels resulting in incomplete ablation localization underneath the diaphragm or close to the liver hilum [5]. As an alternative, computed-tomography-guided high-dose rate brachytherapy (CT-HDRBT) as ablation technique using gamma-irradiation by an iridium-192 source has been developed, with very promising results published, comparable to those of CT-RFA in terms of local tumor control and overall survival [6, 7].

Health care organizations and policy makers are increasingly interested in the costs of health care, leading e.g. to the implementation of diagnosis related groups (DRG) for reimbursement of hospitals in an increasing number of countries [8] and evidenced by many cost-benefit and cost-effectiveness investigations [9].

Activity based cost analysis (ABC), originally invented by Cooper and Kaplan [10], has been used in many service organizations and achieved great benefit when planning activity and budget, because costs can be allocated precisely to their resources and cost factors of activities that form the product [9]. Undisputed, optimal utilization and economic evaluation of limited health care resources is needed and should be based on reliable cost accounting [9].

Therefore, the purpose of this study was to analyse the costs of both ablation techniques and to identify cost factors of the two different procedures. This mainly on the background that CT-RFA is typically performed by interventional radiology alone whereas the newer method of CT-HDRBT has to be performed as an interaction of interventional radiologists and radiation oncologists.

## Materials and methods

### Definition and determination of costs

This study concentrates on the costs of activities in the interventional radiology unit and the radiation oncology department in a German university hospital. The overhead costs for the hospital, wards and the whole radiology unit were not included.

The cost evaluation was based on the recommendations on trial design provided by the International Society for Pharmacoeconomics and Outcomes Research (ISPOR) [11, 12].

The information concerning costs was based on the purchase prices provided by the hospital controlling department and the effective consumption documented in the department’s documentation system (GE Centricity RIS™, Fairfield, CT, USA). The local ethical committee approved the study protocol (EA1/144/11).

*Staff costs* were derived from standard personnel rates provided by the German scientific society (Deutsche Forschungsgemeinschaft, DFG) [13] as costs per minute for the different personnel groups. We assumed 40 working hours/week, 30 days of annual holidays, 15 days mean absenteeism [14] and 9 days of public holidays. We established process models of all steps required in each of the two ablation procedures of any staff member (interventional radiologist, radiation oncologist, medical physics expert, technologists, nurses, Table 1). For each step the duration of involvement was documented in minutes with calculation of mean values. For both treatment techniques, we differentiated between the time for the actual intervention and the time for pre- and post- interventional process steps. The length of the interventional procedure for each patient was calculated retrospectively from the documented DICOM headers of the CT imaging data for therapy guidance. Mean time values (in minutes) for all staff involved in the pre- and post-interventional phases were measured prospectively by documenting the duration of all steps during further ongoing RFA- and HDBRT treatments. The involvement times of the different process steps of all personnel groups were multiplied with their costs per minute. Overall staff costs were then calculated as the sum of costs for the different steps in both treatment pathways.

**Table 1:** Procedural steps and involvement staff of both ablation methods

	CT-HDRBT		CT-RFA	
	Minutes	StaffCode	Minutes	Staff Code
Preparation				
Patient registration	3 ± 0.7	TECH	3 ± 0.63	TECH
Preparation of sterile intervention table	8 ± 1.2	N		
Preparation of sterile intervention table, instruments, and RFA device			12 ± 1.41	N
Patient positioning, unenhanced localizer scan	8^a^ ± 1.6	TECH	12^a^ ± 0.89	TECH
		N		N
Placement of sterile drapes & disinfection	10^a^ ± 1.2	N	10^a^ ± 1.41	N
		TECH		TECH
Review of images, final check-up	5 ± 1.1	INT-RAD	5 ± 0.63	INT-RAD
CT-Intervention				
Induction of conscious sedation	3^a^ ± 0.5	PHYS	3^a^ ± 0	PHYS
		N		N
				TECH
Instrument guidance and positioning of brachytherapy catheters	14^a^ ± 2.4	INT-RAD		
		N		
		TECH		
Instrument guidance and positioning of RFA probe			14^a^ ± 0.89	INT-RAD
				N
				TECH
RFA treatment			*22* ^a^ ± 4.56	INT-RAD
				N
				TECH
Contrast-enhanced CT scan + assessment	5^a^ ± 0.7	INT-RAD	5^a^ ± 0.63	INT-RAD
		N		N
		TECH		TECH
Patient monitoring during intervention and postprocedural scan	19 ± 2.7	PHYS	44 ± 4.73	PHYS
Transfer to brachytherapy suite	10 ± 1.2	PHYS		
		N		
Radiotherapy planning	15 ± 1.5	INT-RAD		
		RO		
		MPE		
Radiotherapy	25 ± 6.2	MPE		
Patient monitoring during radiotherapy	25 ± 6.2	PHYS		
		N		
Postprocedural Management				
Retraction of brachytherapy catheters, tract sealing, and removal of sterile drapes	10 ± 1.5	PHYS		
		N		
Removal of sterile drapes and transfer to recovery room			7 ± 1.26	PHYS
				TECH
				N
Transfer to recovery room	10 ± 1.5	PHYS		
		N		
Monitoring in recovery room	90 ± 11.1	N	90 ± 6.32	N
Documentation and clearance	8 ± 1.4	TECH	8 ± 1.4	TECH
Writing report	5 ± 0.8	INT-RAD	5 ± 0.8	INT-RAD

*PHYS* physician experienced in conscious sedation and for monitoring of the patient, *N* nurse, *TECH* technical assistant, *INT-RAD* interventional radiologist, *MPE* medical physics expert, *RO* radiation oncologist

^a^CT blocked for this time period plus additional 10 min for room cleaning etc. Time needed for intervention and ablation were extracted from institutional PACS data for all patients. All other data were measured in a sample of 5 patients

*Cost for equipment* usage included purchase costs, depreciation, and maintenance costs (Table 2). Prorated costs were calculated on the basis of a 7-year use and linear depreciation according to German tax law for the CT-scanner, afterloading source, RF-heat-generator and the radiation planning workstation. The proportionate costs of the iridium-192 source were calculated based on the service contract with the vendor, which includes a regular source change every 8 weeks and necessary software updates of the radiation planning workstation. Depreciation period for the radiation room was 30 years.

**Table 2:** Expenditures for disposables used in CT-RFA and CT-HDRBT

Material type	n	Costs per unit (in EUR)	
CT-guided RFA			
Set of sterile drapes and coats	1	34	34
Sterile gloves	2	1.48	2.96
Local anesthetic	1	1.56	1.56
RFA probe	1	1460	1460
Contrast medium	1	16	16
Fentanyl	1	0.65	0.65
Midazolam	1	0.35	0.35
Total costs (in EUR)			1515.12
CT-HDRBT			
Set of sterile drapes and coats	1	34	34
Sterile gloves	2 ± 0.43	1.48	3.31 ± 0.64
Local anesthetic	1	1.56	1.56
Puncture cannula	1	22	22
Guide wire	1	23	23
Angiography sheaths	2 ± 0.6	23	42.93 ± 14.46
Brachytherapy catheter	2 ± 0.6	19	35.46 ± 11.95
Fentanyl	1	0.65	0.65
Midazolam	1	0.35	0.35
Gelfoam	1	7.80	7.80
Total costs (in EUR)			187.07 ± 27.05

Total annual use of equipment, maintenance costs, and the length of each specific examination were determined, and based on these data proportionate costs per minute were calculated. Calculated usage of the CT-scanner was 200,000 min per year.

Times of usage of the equipment for the specific patient were measured and included in the calculation.

The *total costs* were computed by addition of costs for the use of equipment, staff costs, and expenditure for disposables and are reported as mean values and standard deviations. For simplification we did not take costs for use of rooms other than the radiation room, for cleaning, and for energy into account.

### Sensitivity and break-even analysis

A model was created calculating theoretical costs per patient depending on a variation of the number of patients treated each year. In this model we assumed a utilization of the devices exclusively for the investigated indication – hepatic tumor ablation. Staff costs, the costs for disposables, and costs for CT were calculated as fixed costs per patient. The annual equipment costs for the RF-generator on the one hand or costs for the afterloading source plus annual depriciation costs of the radiation room on the other were allocated to an assumed annual number of patients. Example values for both methods were calculated and compared for an annual number of 25, 50, 100 and 200 patients treated.

### Patient population

During the intervention of 10 patients (7 men, 3 women; mean age 67.5 ± 5.1) who were treated with CT-guided RFA and 10 patients (8 men, 2 women; mean age 69.3 ± 6.2 years) who underwent CT-HDRBT working steps and time involvement of all participants were recorded. All patients had single HCC target lesions below the size of 3 cm. Uni- and multivariate analysis comparing age, sex, tumor size, tumor location and surround etiology did not show any significant differences between both groups of patients.

Before any ablation of the liver performed at our hospital the lesions had been classified as unresectable by consensual decision of the institutional interdisciplinary tumor board or if patients rejected a possible resection.

The day before the intervention written informed consent was obtained from each patient after extensive explanation of the treatment procedure, possible complications, and alternative treatment options. The local institutional review committee approved the study.

### Method of CT-HDRBT

All patients were treated under conscious sedation using 3–5 mg midazolam (Dormicum™, Hoffmann-La Roche AG, Basel, Switzerland) and 75–200 μg fentanyl (Fentanyl™, Rotexmedica, Trittau, Germany) and monitored by a physician experienced in conscious sedation (PHYS, Table 1) [15, 16]. Dose was adapted individually as required by the patient.

**Fig. 1:**
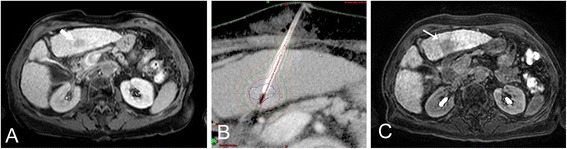
Magnetic resonance imaging scan of a 69-year-old patient with liver cirrhosis and hepatocellular carcinoma in liver segment III (**a**, arrow). The tumor was treated by CT-guided high-dose rate brachytherapy with a single catheter and a tumor-enclosing target dose of 20 Gy (**b**). Six weeks later, complete tumor ablation is indicated by a surrounding lack of uptake of hepatocyte-specific contrast medium (Primovist™, Bayer Healthcare, Leverkusen, Germany) (**c**, arrow)

Procedure of CT-HDRBT has been explained extensively elsewhere [6, 15, 16], however, we summarize briefly the working steps of the involved staff: After a spiral scan of the upper abdomen, performed by a radiological technician (TECH), a nurse (N) covered the patient with sterile drapes and disinfected the puncture site. After additional local anesthesia and a point-shaped skin incision, 6-French angiographic sheaths (Cordis Avanti + 6F Sheath™, Cordis Cooperation, Bridgewater, NJ, USA) were inserted into the hepatic target lesions by an interventional radiologist (INT-RAD) using the Seldinger’s technique (17 Gauge (G) Puncture Needle, KLS Martin, Umkirch, Germany, and a 145 cm stiff guidewire, Amplatz Super Stiff™, Boston Scientific, Natick, MA, USA ). They served as stabilization devices for the closed-ended 6F afterloading catheters (Primed, Halberstadt, Germany) which were introduced subsequently. Catheter positions in relation to the hepatic lesions were depicted on a contrast-enhanced CT scan that was also used for further treatment planning on a three-dimensional (3D) radiation planning workstation (Brachyvision™, Varian Medical Systems, Palo Alto, CA, USA; Fig. 1). A medical physics expert (MPE), a radiation oncologist (RO) and the interventional radiologist planned subsequent radiation. The MPE coordinated the handling and insertion of the iridium-192 source (Gammamed 12i™, Varian Medical Systems, Palo Alto, CA, USA). Patients were transferred to the brachytherapy suite under conscious sedation, monitored and accompanied by a physician and a nurse. During retraction of the catheters, the puncture tracts were sealed with resorbable thrombogenic material (Gelfoam™, Pfizer Inc., NY, USA) to minimize the risk of bleeding. After transfer back to the department’s recovery room patients were surveyed for approx. 90 min before being transferred to the department’s ward.

### Method of CT-RFA

**Fig. 2:**
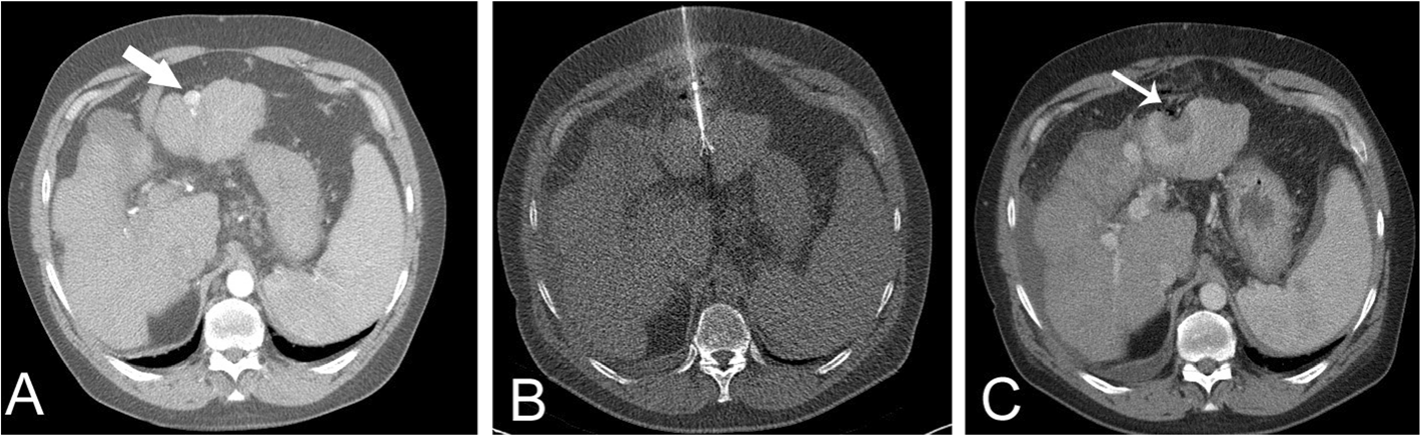
A 65-year-old patient with cirrhosis and a hypervascularized, subcapsular hepatocellular carcinoma in liver segment III (**a**, arrow). The tumor was ablated in a single session with 110 W over 15 min (**b**). The contrast-enhanced control scan obtained immediately afterwards shows complete necrosis of the ablated area with a hypervascular rim (**c**, arrow)

All patients were treated under conscious sedation using fentanyl and midazolam monitored by a physician experienced in conscious sedation (PHYS, Table 1), in the same way as conscious sedation was induced during CT-HDRBT. After a spiral scan of the upper abdomen including the whole liver, performed by a radiological technician (TECH), a nurse (N) covered the patient with sterile drapes and disinfected the puncture site. Local anesthesia was administered in the region of the planned puncture site (Xylonest 0.5 %, AstraZeneca, Wedel, Germany). After a point-shaped stab skin incision, the ablation probe was advanced into the hepatic tumor under CT fluoroscopic guidance (RITA Starburst™, RITA Medical Systems, Fremont, CA, USA) by an experienced interventional radiologist (INT-RAD). Tumors were ablated according to the vendor’s recommendations using 90–150 W (Generator Model 1500, RITA Medical Systems, Mountain View, USA) [17]. After ablation of the target lesion, the probe was switched to the tract ablation mode to ablate the tract during retraction of the probe. To assess whether complete ablation of the target lesion was accomplished, a contrast-enhanced spiral CT scan was performed (120 ml Ultravist™ 370, Bayer Healthcare, Leverkusen, Germany, Fig. 2). A nurse monitored all patients for 90 min after the interventions in the recovery room before they were transferred to the department’s ward.

## Results

Workings steps and staff involved for CT-HDRBT and CT-RFA procedures and their durations are listed in Table 1. Calculated staff costs per minute (in EUR) were 0.874 € for a physician, for the medical physics expert 0.516 € and 0.455 € for a nurse or technologist. Based on the staff involvement times presented in Table 1, average staff costs summed to 252.80 ± 19.65 € per patient for CT-HDRBT (mean total duration: 277 min) and to 211.36 ± 15.42 € per patient for CT-RFA (mean total duration: 242 min).

Counting as variable disposable costs, the mean number of brachytherapy catheters per patient was 2 ± 0.6 for CT-HDRBT treatment and 1 probe per patient for RFA treatment. Expenditure for disposables summed up to 187.07 ± 27.05 € per patient for CT-HDRBT and 1,515.12 € ± 0 per patient for RFA (Table 2).

All these costs were independent from the number patients treated.

Proportionate costs for equipment were strongly dependent on the number of patients treated annually by each method (Table 3). Fix costs which had to be allocated to the number of patients were 74,019.05 € for CT-HDRBT and 3,098.57 € for CT-RFA.

**Table 3:** Costs for equipment for CT-HDBRT and CT-RFA

	Siemens somatom definition AS	RITA RF generator	Varian medical gammamed + planning workstation	Radiation room
Purchase Costs	670,000	19,940	135,000	400,000
Annual Maintenance	45,000	250	36,400	5,000
Depriciation Period in years	7	7	7	30
Annual Usage in min	200,000	TBD^a^	TBD^a^	TBD^a^
Proportionate Equipments Costs				
	CT-RFA	CT-HDBRT		
1 patient / year	3,098.57	74,019.05		
25 patients / year	123.94	29,60.76		
50 patients / year	61.97	1,480.38		
100 patients /year	30.99	740.19		
200 patients /year	15.49	370.10		

^a^ *TBD* To be determined. The annual usage was assumed to be variable, as indicated by the proportionate equipment costs in the lower part of the table

Total costs per intervention for the 25, 50, 100 or 200 patients treated annually were 1,909.52€, 1,847.55 €, 1,816.57 € and 1,801.07 € for CT-RFA whereas 3,442.85 €, 1,962.47 €, 1,222.27 € and 852.18 € were calculated for CT-HDRBT.

**Fig. 3:**
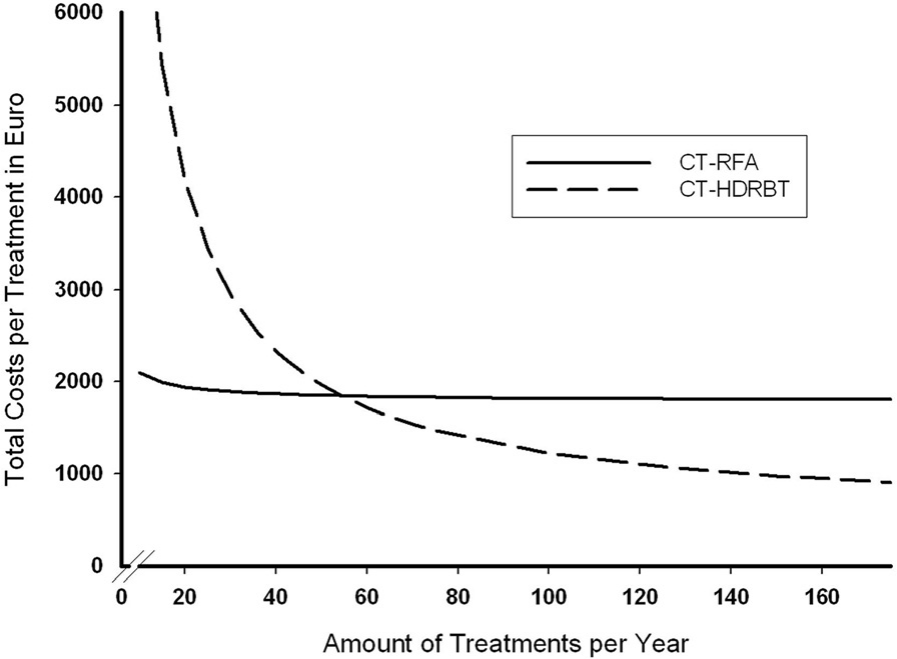
Results of a sensitivity analysis comparing costs of CT-HDRBT and CT-RFA in relation to the number of patients treated with either method per year. The break-even is reached at 55 treated patients per year, when calculated costs of CT-HDRBT fall below the costs of CT-RFA. At this point, calculated costs for CT-RFA are 1,841.92 € and 1,827.89 € for CT-HDRBT, and costs for CT-HDRBT decrease further as the number of treated patients increases, whereas costs for CT-RFA remain nearly stable

Figure 3 illustrates these different cost characteristics of both methods, with a break-even for CT-HDRBT at 55 patients.

## Discussion

Local tumor ablation has become an important part in the treatment of oncologic patients, especially patients with HCC. Various techniques for local therapies have been established, but only few cost identifications have been performed [18] and this is the first one using an ABC analysis.

ABC accounting was originally designed for use in industry and service production units, and has been found to serve well in health care service units [9, 19]. RFA and CT-HDRBT are competing ablation methods for the treatment of HCC. Both treatments have become established in clinical routine and have shown to be safe and effective in terms of local tumor control and prolonging patient survival [6, 15, 20, 21].

In any health care system resources are limited, and therefore the most cost-effective method should be used when there are two competing methods with comparable clinical outcome, as far as single arm studies in the literature can be compared. However, no randomized trial comparing both methods has so far been performed to investigate therapeutic differences as direct comparison.

We found in our model that the more complex method of HDBRT can be performed at lesser costs than the well-established method CT-guided RFA when more than 55 patients are treated annually. As a main costs advantage, CT-guided brachytherapy necessitates only standard disposable material that is much lower in price than dedicated RFA-probes, which have a relatively high price and are only suitable for single use. This fact has never been reported before and is important to consider, as there is high need for cost-saving procedures in the increasingly more expensive healthcare system.

The relatively high annual maintenance costs of 36,400 € for the Iridium-192 source and the costs for the radiation room are compensated when treating 55 patients or more with CT-HDBRT annually.

From our own experience CT-HDBRT is feasible in more patients, as also big tumors or tumors close to sensitive structures can be treated where CT-RFA is limited [15, 20, 22].

The advantage of CT-guided RFA is that it can be performed in one single room: planning of the procedure, image-guided needle placement, ablation, and control of success can be performed consecutively in one session and in a single room within the CT scanner. As a result, CT-RFA has a higher demand of CT in room time which leads to higher equipment costs. Orientation during the intervention is typically limited as it is based on CT fluoroscopy images where the shape of the ablation needle defines the ablation zone. CT-HDRBT requires a more interdisciplinary approach and more staff involved, as it is typically a close cooperation of radiation oncologists and interventional radiologists. Interstitial brachytherapy in afterloading technique has to be performed in a dedicated radiation room with special structural requirements [23], making equipment costs higher compared to CT-RFA.

Given the more complex approach of CT-HDRBT as Table 1 shows, it is somewhat surprising that CT-HDRBT is can be performed at lower price than CT-RFA. This difference is mainly attributable to the high costs for disposables, namely the RFA probe, which by far tops the costs for the sheath and brachytherapy catheters (Table 2). Costs for other disposables are nearly the same, as both interventions are performed in comparable settings.

It is not surprising that staff costs were higher for CT-HDRBT than for CT-RFA as additional specialists (medical physics experts and radiation oncologists) from the radiation oncology department had to be consulted and transfer times between different rooms had to be included. In the setting of our hospital the interventional radiologist has the qualification to perform brachytherapy which is approved by the responsible medical association, but to make data applicable for other institutions we included a radiation oncologist in our calculations.

Fixed annual costs are higher for CT-HDRBT, as the iridium source has to be changed every eight weeks due to the half-life of 74 days of iridium-192 and the radiation room has to depreciated [23]. During this period, any number of patients can theoretically be treated with one source, so the proportionate costs for the source strongly depends on the number of patients treated per year, as we could show in the sensitivity analysis (Fig. 3). In general, we perform local ablations of hepatic malignancies, both CT-HDRBT and CT-RFA, in conscious sedation [16]. Only in rare cases we do use general anesthesia, mostly when patients explicitly prefer this option. The additional anesthetic team would raise staff and equipment costs, but as this approach is rare with both treatment options, we did not include these costs in our calculation.

With regard to the prices for disposables, our hospital profits from overall low prices as we are a part of a large purchase network of private and university hospitals. Therefore, our purchase prices do not differ significantly in relation to the amount of items purchased. Therefore, the prices for disposables did not vary with regard to the amount of consumed disposables. However, small hospitals may have to pay much higher prices for high priced disposables like RFA probes. In relative terms the use of a therapy option like HDBRT with low costs for disposables would especially be useful for smaller hospitals in the case they do not have the opportunity to get low prices for high priced disposables like RFA probes as long as their amount of consumed items is low. A break-even for a new treatment option like HDBRT may then be reached even with lesser patients than in our clinic as we already profit from low purchase prices for RFA probes. In Germany, the health care system has a dual financing [24]: investment costs for real estates and large equipment are borne by the federal states, and the costs directly associated with the treatment (e.g., staff costs and costs for disposables) are covered by health insurance providers. For this reason, cost for rooms are typically not in the focus of health care providers but we had to include the radiation room in our calculation as it is mandatory for tumor therapy in afterloading technique.

Our study has limitations: The sample size of patients treated by RFA was limited. This is because CT-HDRBT is preferred to CT-RFA in our department – mainly because of the subjectively higher accuracy. Reported prices and staff costs are from the year 2015, and participation of different team members were reflected as they are typical in the German health care system. In other countries, certain activities might also be performed by other staff groups.

We are aware that both the costs for medical devices and personnel and also the reimbursement for healthcare services differ significantly between different countries throughout Europe and worldwide. However, as our focus was on relative and not absolute costs for the two different techniques for the treatment of HCC, we expect the main result of our cost comparison to be valid in many different countries.

We did not take into account “overhead” costs like costs for energy, administration, and cleaning and other rooms beside the radiation room. The exact assessment of these costs usually is very difficult and, as they are mainly the same for both procedures, we did not expect them to influence the overall result.

In this context, it is arguable why we assumed an exclusive utilization of the RFA generator, the radiation therapy planning station and the brachytherapy room. We were aware the RFA generator and the brachytherapy device can not only be used exclusively for the two different HCC therapy techniques but for many other indications such as radiofrequency ablations of osteoid osteomas and the brachytherapy of gynaecological tumors. This underlines the importance of interdisciplinary cooperation for therapeutic decision making [25] and an economic and synergetic use of the equipment.

However, this shared usage may be a major confounding variable as especially the costs for CT-HDRBT differ substantially depending on the number of patients treated. We could not expect that other clinics and especially providers that have to consider to begin with HDBRT will treat the very high number of about 480 patients / year as we do. Therefore, our own low costs for HDBRT may be misleading for the reader. With the aim to provide a more realistic overview of the own costs also for clinics considering to start with both procedures, we assumed that the RFA and HDBRT equipment (which also includes the radiation room) would be exclusively acquired for the two treatments.

In contrast, most clinics nowadays have CT scanners available which usually are intensively used for diagnostic purposes and the use for CT-guided interventions is mostly only in a much lesser proportion. Therefore, we did not assume an exclusive utilization of the CT scanner for our two CT-guided therapy options, but assumed a proportionate utilization of the overall 200.000 min per year.

Finally, the prices quoted in this paper are specific to our hospital and may differ for other hospitals. However, the idea of the study was to give the reader a model for a quick analysis of the own costs for the standard treatment option of RFA and a new technique like CT-HDBRT. Personal differences in prices may easily be adapted by the reader. In this context, we did not consider “hotel” costs in our analysis. For both RFA and HDBRT the mean duration of a hospital stay is about 2–3 days, and it would be easy to calculate the hospitality costs which are about 450 EUR per patient and day. As we performed a cost comparison with relative costs, we did not expect a difference in these costs for both procedures and did not take “hotel” costs into account. We also expected a significant variation in reimbursement for both procedures in different countries. This is why we focused exclusively on a model to evaluate the own costs for both procedures which can then be compared with the individual reimbursement scheme.

## Conclusion

From the provider’s perspective, the costs of CT-HDRBT of liver lesions are much lower than those of CT-RFA treatment as soon as at least 55 patients are treated annually. This is mainly due to relatively high fixed cost per CT-HDRBT treatment but much lower costs for disposables compared with CT-guided RFA treatment.

## Abbreviations

ABC: activity based cost analysis
CT: computed tomography
HCC: hepatocellular carcinoma
HDRBT: high dose rate brachytherapy
INT-RAD: interventional radiologist
N: nurse
PHYS: physician
RFA: radio-frequency ablation
RO: radiation oncologist
TECH: radiological technician
